# The Development of an Intramuscular Injection Simulation for Nursing Students

**DOI:** 10.7759/cureus.12366

**Published:** 2020-12-29

**Authors:** Julia Micallef, Artur Arutiunian, Adam Dubrowski

**Affiliations:** 1 Health Sciences, Ontario Tech University, Oshawa, CAN

**Keywords:** intramuscular injections, simulation, training

## Abstract

Intramuscular (IM) injections are preferred over subcutaneous injections for administering medicine such as epinephrine and vaccines as the muscle tissue contains an increased vascular supply that provides ideal absorption of the drug being administered. However, administering an IM injection requires clinical judgment when choosing the injection site, understanding the relevant anatomy and physiology as well as the principles and techniques for administering an IM injection. Therefore, it is essential to learn and perform IM injections using injection simulators to practice the skill before administering to a real patient. Current IM injection simulators either favor realism at the expense of standardization or are expensive but do not provide a realistic experience. Therefore, it is imperative to develop an inexpensive but realistic intramuscular injection simulator that can be used to train nursing students so that they can be prepared for when they enter the clinical setting.

This technical report aims to provide an overview of the development of an inexpensive and realistic deltoid simulator geared to teach nursing students the skill of IM injections. After development, the IM simulators were tested and validated by practicing nurses. An 18-item survey was administered to the nurses, and results indicated positive feedback about the realism of the simulator, in comparison to previous models used, such as the Wallcur® PRACTI-Injecta Pads (Wallcur LLC, San Diego, CA). Feedback to improve the density of the simulator as well as the shape and size to make it a more realistic experience was provided.

## Introduction

Intramuscular (IM) injections are the preferred route of administration for medicine in clinical settings. This preference over subcutaneous injections, which is a drug administration route that is right below the dermis and epidermis layer, is due to the increased vascular supply that is available in muscle tissue, providing ideal absorption of the drug being administered [1]. There are challenges if IM injection misses the muscle and is not deposited in the fat, resulting in some or all of the medicine being wasted. Moreover, side effects can be swelling, redness, tingling or numbness, drainage at the injection site, prolonged bleeding if a blood vessel is pierced, and possibly some pain. Therefore, it is vital to utilize injection simulators to practice the skill away from the patient. Studies show that there is only a 32% to 52% success rate in performing IM injections, and the remaining unsuccessful administrations can result in adverse health and psychiatric effects on their patients. It has been shown that further education and training in the simulation of the IM injections can increase the success rate to 75% [1,2]. The use of an IM injection simulation to practice this skill will provide an immersive learning strategy to improve competency and confidence when performing this skill on an actual patient [3].

Currently, there are two types of simulators being used: animal and synthetics. Animal models are realistic, but there are issues with the ethics of using these: standardization, health, and safety. On the contrary, synthetic models are good alternatives; however, they are costly, often lack realism, and do not provide the variability that is often needed for learning [4]. With the advent of additive manufacturing, such as 3D printing, we are now able to design and build inexpensive, realistic, and variable synthetic simulators [5-8]. Developing inexpensive IM injection simulations directed to nursing students will allow the aspiring nurses to become well acquainted with the skill before entering the clinical setting. Furthermore, this will generate the confidence and proper technique to allow the best experience for the patient [9,10].

This technical report will provide an inexpensive and realistic simulation for nursing students to perform IM injections successfully. Currently, there is no technical report available describing the manufacturing process to develop an IM injection simulation that is simple yet provides a realistic outcome at a small cost. We aim to describe and share the development of a realistic, customizable, and inexpensive IM injection model, along with the assessment of the perceived realism and potential for use as an educational tool of the model. The outcome will be that the inexpensive and realistic IM simulation will provide nursing students with a tool that they can utilize to practice this necessary skill, so they are better prepared for when they enter the clinical setting.

## Technical report

Context

The IM injection simulators were designed to be used as a training tool for pre-licensure nursing students and to train first responders. One could also consider this task trainer as a low-cost tool for the maintenance of skills for in-hospital nursing staff. Because the manufacturing is based on off-the-shelf supplies available in local stores and on-line (e.g., silicone), these simulators can be also used for injection skills development and upkeep in rural and remote areas.

Inputs

We have used the following materials to build the IM injection simulators: Jumbo cupcake trays, stirring sticks, mixing containers, Dragon Skin^TM^ NV 10 (silicone base), Slacker^TM^ (silicone softener), Silc-Pig^TM^ (color) (Smooth-On, Inc., Pennsylvania), absorption sponge, and Ease-Release spray.

Process

Construction of the Simulator

**Figure 1:**
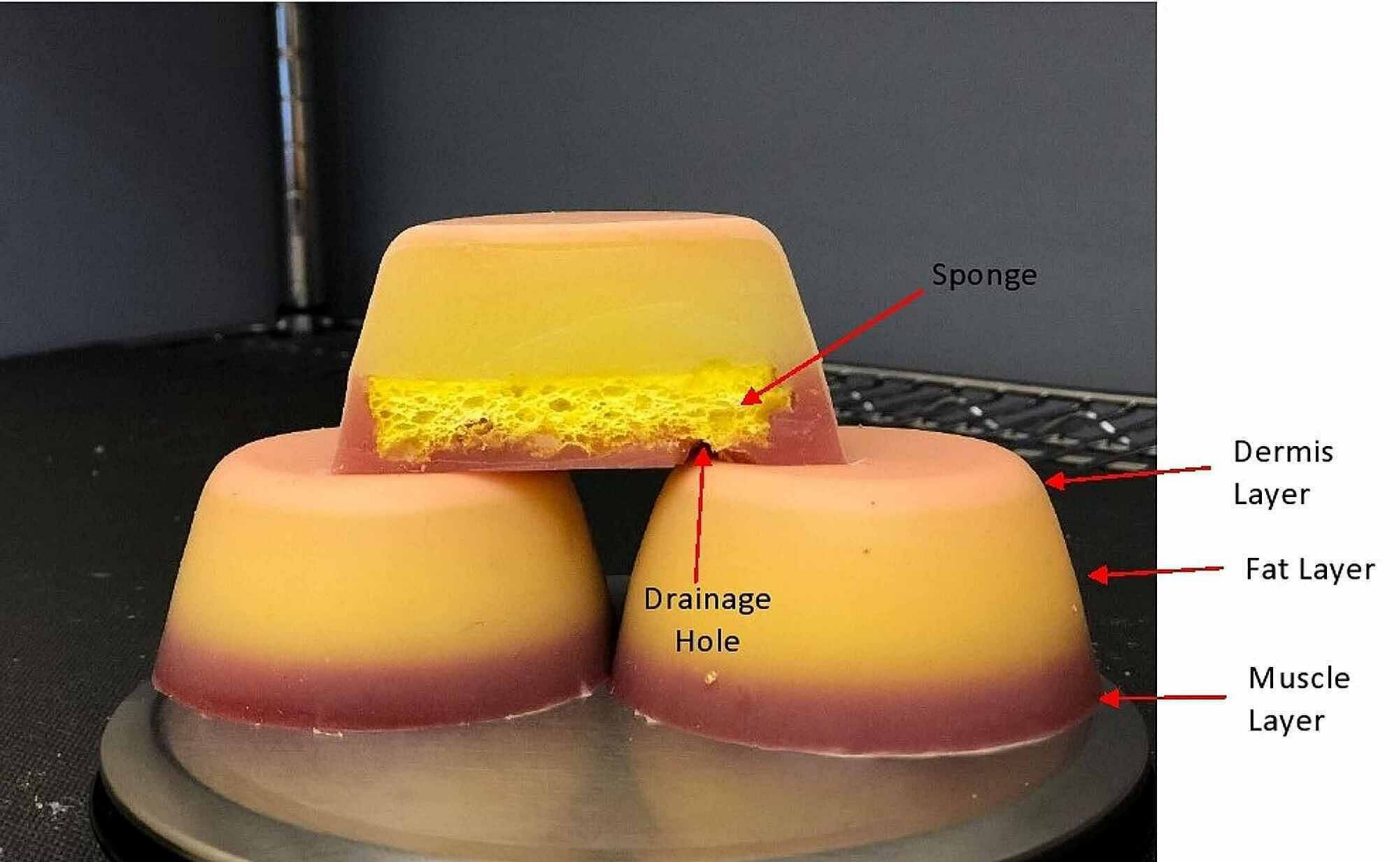
Side and cross-sectional view of IM simulators IM, Intramuscular.

Jumbo cupcake trays with a diameter of 3.5 inches (89 mm) were used as our molds for the IM injection simulators. Smooth-On Dragon Skin™ 10 NV silicone and Silc-Pig™ neutral tones were used to create the thin dermis layer, using a 1-part A:1-part B mix ratio. It was set to cure for 75 minutes without a heat source but can be placed at a temperature of 75˚C-85˚C to allow for a 10-minute curing time. To create a less stiff fat layer, Smooth-On Dragon Skin™ 10 NV was used in addition to Slacker™, creating a ratio of 1-part A:1-part B:2-part slacker. This mixture was colored yellow using Silc-Pig™ and poured to fill approximately half of the cupcake trays. The addition of the slacker required a longer curing time of approximately four hours without the heat source and one hour with the heat source. A sponge cut to a 2.5 inch (63.5 mm) diameter was placed atop the fat layer to absorb the injected liquid. The muscle layer was created using 1-part A:1-part B:1-part slacker to generate a stiffer layer than the fat but not as stiff as the dermis layer. The muscle layer was colored red using Silc-Pig™ and poured to fill the remainder of the cupcake tray and cover the sponge. This step took approximately two hours to cure without the heat source and 45 minutes with the heat source. The heat source used was the Ultimaker S5 heated print bed set at 75˚C-85˚C. One-inch (25.4 mm) pieces of plastic straws were added to the muscle layer before it was left to cure so that when finished, the straw can be removed, leaving behind a drainage hole for the liquid that will be absorbed in the sponge. Once completely cured, each simulator was removed from the mold (Figure 1).

**Figure 2:**
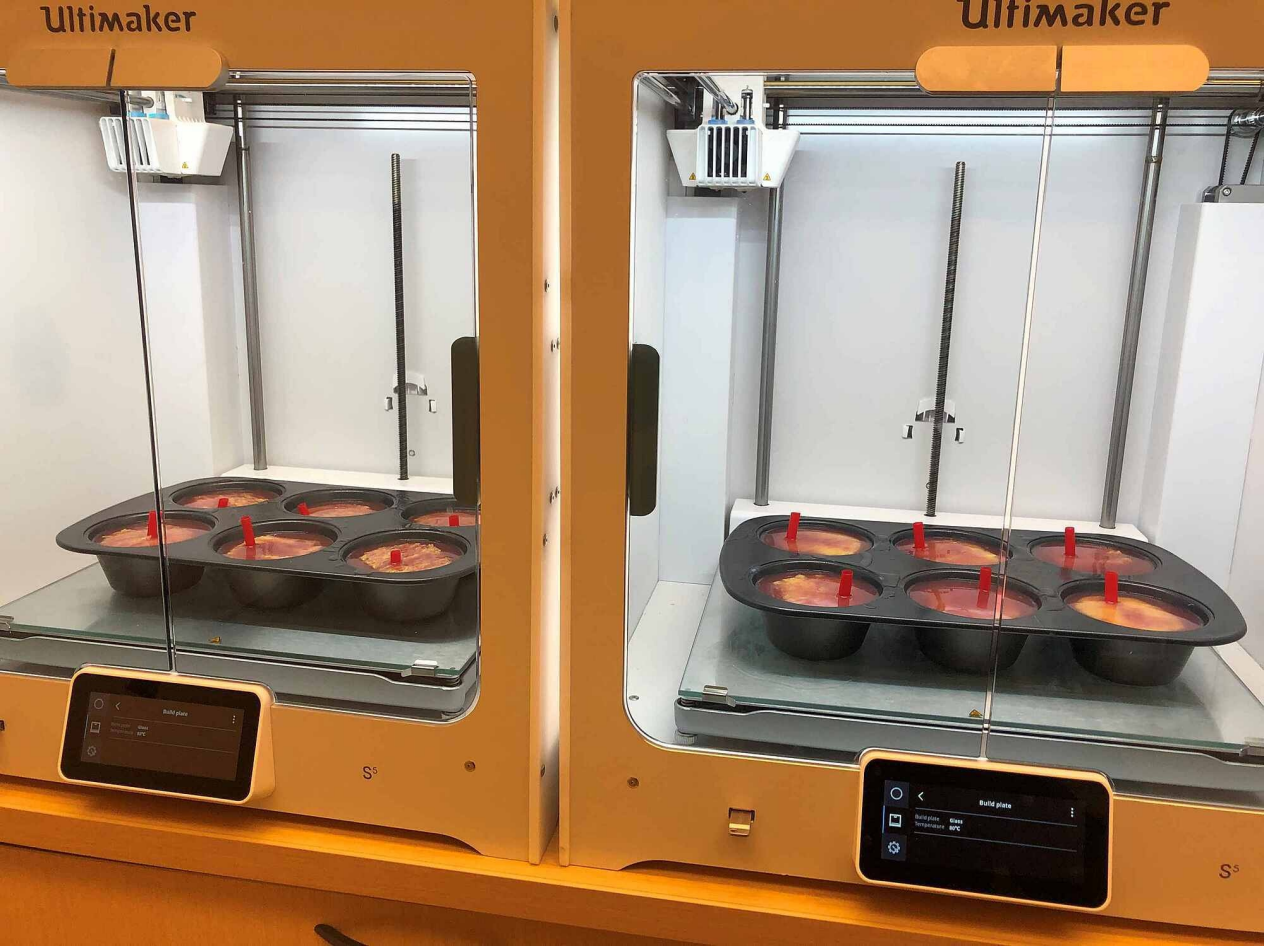
Using the Ultimaker S5 heating bed set at 75˚C for the final curing step

The price to make a single IM injection simulator was calculated to be CAD$10.27 (Table 1). This table shows the cost breakdown for manufacturing 140 simulators used during a curricular session at Ontario Tech University, Canada. The manufacturing took approximately two to six hours with two students working on it, and the speed was dependent on the availability of a heat source for curing steps. We have placed the simulators inside of a three-dimensional (3D) printer with a temperature set to 75˚C in between each curing step to speed up the process (Figure 2).

**Table 1:** Cost breakdown of the materials (in CAD) needed to produce 140 IM injection simulators IM, Intramuscular; CAD, Canadian dollars.

Material	Amount used for 140 pcs	Price per item	Total cost (tax in)
Slacker^TM^ (silicone softener)	8634 g	$728.00/40lb	$346.45
Dragon Skin^TM^ NV 10 (silicone base)	12,994 g	$312.00/16lb	$558.6
Absorption sponge	8 packs (9 sponges in each)	$10.48/pack	$83.84
Silc-Pig^TM^ (color)	1 sample pack	$47.90/pack	$47.9
Ease-Release spray	1 can	$19.36/can	$19.36
Molds	3 trays	$9.03/tray	$27.09
Stirring sticks	2 packs	$3.92/pack	$7.84
Mixing cups	1 pack (50 pcs)	$9.03/pack	$9.03
Table cover	1	$1.41	$1.41
Test syringe	3	$0.85	$0.85
Isopropyl alcohol (99%)	500 ml	$8.46	$8.46
Latex-free gloves	100 pcs box	$13.55	$13.55
Mixing containers (2 L)	7 pcs	$3.92/2 pcs	$14.02
Labor	20 hours	$15/hour	$300
		FINAL TOTAL	$1438.40
		COST PER IM SIMULATOR	$10.27

Design Feedback

The initial feedback received on the simulator’s realism and usefulness for training was provided by volunteer nurses that practice in-hospital settings, specifically as an educational product quality improvement initiative. The development team contacted the Clinical Practice Leader of the Nursing Professional Practice Council to ask the participating nurses to try the simulators. All participants were informed that the development of this simulator is not related to their work and professional development activities and that their participation is voluntary and anonymous. They were also told that they would be sent an anonymous Google form’s link with a survey to provide feedback on the simulator. The participants did not know the development team, and none of the members of the development team knew any of the participants. We asked 20 nurses during their monthly orientation session to perform IM injections on the new simulator and provide us with feedback on possible improvements. We provided 20 identical IM injection simulators, one for each nurse, and each was paired with a one-inch (25.4 mm) long, 22-gauge needle, alcohol wipe, and water to act as the injecting liquid. Each volunteer nurse performed the injection a minimum of three times using the simulator and the provided tools. After that, they were sent a link to a survey via Google Forms to provide us feedback that assessed the realism of the IM injection pad, how it compared to previous models, and possible improvements to the simulator. The survey was based on a prior publication by Goudie et al. (2018) in which the authors tested the efficacy of their developed silicone model for perineal repair suturing [10]. There were total of 18 questions that included comparing the effectiveness and realism of previous models to the newly developed model, questions assessing whether they would recommend this simulator as an educational tool for nursing students, rating on a scale of 1-10, the realism of various components of the model, and suggestions for further improvements (Table 2). We had a total of 11 volunteer nurses fill out the survey anonymously, giving us a 55% response rate.

**Table 2:** Questions from the survey filled out by hospital-based practicing nurses

Question number	Question
1	What is an intramuscular injection simulation that you have used in the past, if you have used any?
2	If you have previously used another intramuscular injection simulation, how effective was that simulator in increasing your competency on the procedure?
3	If you have previously used another intramuscular injection simulation, how effective was that simulator in increasing your confidence in performing the procedure?
4	If you have used other intramuscular injection simulations before, how does this injection pad compare to the previous model?
5	Please elaborate your answer for question 4.
6	Would you recommend the use of the new injection pad to assist with training and education of intramuscular injections for students?
7	To serve as a practice simulation of an intramuscular injection for nursing students, please rate how realistic the color of the injection pad is.
8	To serve as a practice simulation of an intramuscular injection for nursing students, please rate how realistic the thickness of dermis layer of the injection pad is.
9	To serve as a practice simulation of an intramuscular injection for nursing students, please rate how realistic the softness of dermis layer of the injection pad is.
10	To serve as a practice simulation of an intramuscular injection for nursing students, please rate how realistic the softness of fat layer of the injection pad is.
11	To serve as a practice simulation of an intramuscular injection for nursing students, please rate how realistic the softness of muscle layer of the injection pad is.
12	How would you improve the new injection pad?
13	Did you experience any difficulties or discomfort while using the model?
14	Can you feel the difference between the different layers (dermis, fat, and muscle)? If not, is it important that there is a definite distinction, or is it good as is?
15	Was the sponge effective in absorbing the liquid?
16	Was the drainage hole effective in draining the liquid from the injection pad?
17	Do you have any additional comments you would like to note?
18	How often do you perform intramuscular injections in clinical settings?

Outcome

Simulator

The IM injection simulator includes a tough dermis layer, a soft fat layer in the middle, and a mid-level stiffness muscle layer. In between the fat and muscle layer lies a sponge to absorb the liquid that will be used during the injection as well as a drainage hole in the muscle layer to provide easy drainage from the sponge. This simulator has been tested in-house as a part of the development cycle to withstand thousands of injections to provide long-time usage with minimal damage.

Assessment and Feedback

The survey data are considered ordinal data. Although the debate is open, whether this type of data can be interpreted using parametric or non-parametric statistics [11,12]. Since our participant numbers are low and because the point of the analyses was to inform the design, rather than to provide evidence of validity, we decided not to use inferential statistics but instead present the data in the form of descriptive statistics. As such, data are presented both as frequencies of distribution as per each question as well as mean and standard deviations. These dual representations of data enable a more informed discussion that leads to informing our designs. Of the 11 nurses that filled out the survey, nine agreed that the new IM injection simulation was more realistic than any previous IM simulator that they have used (Table 3). The volunteers noted from question 5 of the survey that the IM injection simulator mimics the feel of actual skin as well as muscle. It was also noted that the varying densities of the IM injection simulator added to the realistic experience. The previous models used to practice IM injections were the PRACTI-Injecta Pads® (Wallcur LLC, San Diego, CA), a mannequin, and injection pads filled with fluid. Additionally, all the experts that filed out the survey agreed that they would recommend the new IM simulator to assist in the training and education of nursing students with 54.5% strongly agreeing and 45.5% agreeing (Table 3).

**Table 3:** Response frequency of questions 2-4 and 6 from the survey given to hospital-based practicing nurses Refer to Table 2 to match the question number with the actual question.

Question number	Not at all effective	Slightly effective	Neutral	Effective	Very effective	Total responses	Mean	Standard deviation
2		4	2	3		9	3	1
3		4	2	2	1	9	2.25	1.2583057
	Less realistic	Neutral	More realistic					
4			9			9	9	N/A
	Strongly agree	Disagree	Neutral	Agree	Strongly agree			
6				5	6	11	5.5	0.7071068

When addressing the realism of the IM injection simulation, the responses that rated the realism of the various components of the IM injection simulation on a scale of 1 to 10 can be seen in Table 4.

**Table 4:** Response frequency of questions 7-11 from the survey given to the hospital-based practicing nurses to rate the realism of the IM injection on a scale of 1 (not realistic) to 10 (very realistic) Refer to Table 2 to match the question number with the actual question. IM, Intramuscular.

Question Number	1	2	3	4	5	6	7	8	9	10	Total responses	Mean	Standard deviation
7						2	1	4	3	1	11	2.2	1.3038405
8						1	2	4	3	1	11	2.2	1.3038405
9					1	1	2	3	4		11	2.2	1.3038405
10							4	3	4		11	3.666667	0.5773503
11							3	4	4		11	3.666667	0.5773503

## Discussion

We aimed to describe and share the development of a realistic, customizable, and inexpensive IM injection model, along with user-based feedback of the perceived realism and potential for use as an educational tool of the simulator model. Most of the feedback we received from the hospital-based practicing nurses was positive and helpful for additional improvements. The main areas of concern were the densities of the layers being too stiff so that the experience does not simulate performing the IM injection simulation on a patient. Additionally, the nurses noted that the shape of the simulator should be molded to be closer to that of the human body for a more realistic simulation. However, despite the critiques, all the volunteer nurses unanimously agreed that this new IM injection simulation is more realistic than any other previous models they have used.

Therefore, based on the feedback, we believe that the simulator, in its current state, is an acceptable and feasible solution for rural and remote areas. This is because the perceived realism and educational purposes were scored highly, and the simulator can be made easily by untrained personnel. For example, rural and remote training facilities can team up with local high-schools or community centers for seniors to engage in collaborative manufacturing. For the elderly, literature shows that engagement in leisure activities has been claimed to be highly beneficial as well [13]. Practicing such activities may help older adults to preserve cognitive function, physical function, and mental health, and thus to contribute to successful aging [13]. For youth, engagement in such activities, especially in rural and remote areas, may provide not only an alternative set of extracurricular activities but also may allow students to be exposed to health care education and practice, leading them to consider these as alternative career choices.

More urban centers may also benefit from more realistic simulators. Consequently, the next step in our research will be to improve the realism of the IM simulator. To start this process, we have developed and provided an open-access 3D printable model of a shoulder (this will mimic the deltoid site that is commonly used for IM injections) that can be used as a substitute to the cupcake trays as molds [14]. These 3D printed models can be used as molds to form more anatomically correct models. Although this option provides a more realistic representation of the injection surfaces, it increases the costs and manufacturing logistics. Specifically, the increased costs are associated with the printing materials (around CAD$3.26 per model, one-time cost only) and the increase of silicone used to approximately CAD$10-15 per mold. Logistically, this option requires access to 3D printers to manufacture the mold.

## Conclusions

Silicone IM injection models are a cost-effective and more realistic approach to aid in the education of nursing students. With the feedback, we can modify the model to create an even more realistic experience that will allow nursing students to get hands-on experience in IM injections to be better prepared when entering the clinical setting.
